# Serological and molecular survey of *Toxoplasma gondii* infection and associated risk factors in urban cats in Kunming, Southwest China

**DOI:** 10.3389/fvets.2024.1393236

**Published:** 2024-06-18

**Authors:** Chunli Yang, Songhao Liu, Cong Tao, Jing Yu, Mengping Yang, Lijuan Guo, Liya Bao, Xiaobing Li, Jing Yang, Kangfeng Jiang

**Affiliations:** ^1^College of Veterinary Medicine, Yunnan Agricultural University, Kunming, Yunnan, China; ^2^Fujian Provincial Key Laboratory for Prevention and Control of Animal Infectious Diseases and Biotechnology, Longyan University, Longyan, Fujian, China

**Keywords:** *Toxoplasma gondii*, cat, seroprevalence, molecular investigation, risk factor, zoonosis

## Abstract

*Toxoplasma gondii* (*T. gondii*) is a worldwide zoonotic parasite that can infect almost warm-blood animals, including humans, which seriously affect the health of host. Cats are known to be the only definitive host of *T. gondii* and continuously excrete highly infectious oocysts. This parasite carried by the companion animals leads to a great public health risk. However, there is little information on epidemiology of *T. gondii* in urban cats in Kunming, Southwest China. In the present study, a total of 231 serum and fecal samples were collected in Kunming aera, and then seroprevalence of *T. gondii* IgG antibodies in serum and molecular investigation in feces were analyzed to elucidate *T. gondii* infection in urban cats. The results revealed that 168 of 231 cats (72.7%) were positive for *T. gondii* antibodies, and 1 of 74 cat feces (1.4%) also showed a positive PCR for *T. gondii* DNA. The positive fecal sample was sequenced and then phylogenetically analyzed, and the isolate of *T. gondii* in the present study was closely related to *T. gondii* strain CN. In addition, the food, water and age of cats were identified as the risk factor for seropositivity. Overall, our findings indicate the widespread occurrence of *T. gondii* infection in urban cats in Kunming, Southwest China and identify food, water and age are the risk factors associated with *T. gondii* infection, which can provide effective information for developing strategies to prevent and control this zoonosis.

## Introduction

*Toxoplasma gondii* (*T. gondii*) is an obligate intracellular parasite with wide worldwide distribution and host range that can infect almost all warm-blooded animals, leading to severe zoonosis (1). This parasite infects one-third of the world population and numerous animals, which raises public health concern (2, 3). The ingestion of oocyst-contaminated water and undercooked food containing cysts are the main sources of *T. gondii* infection. As definitive hosts of this parasite, cats play an important role in life cycle and spread of *T. gondii* (4). Sexual reproduction of the parasite occurs in the intestinal epithelium of cats, resulting in the formation of oocysts. The oocysts eventually are excreted in cat feces, contaminating the environment and causing infections in other animals (5, 6). Primary infection is often subclinical, whereas in immunocompromised hosts, the latent infection can be activated and develop into life-threatening toxoplasmosis, including lethal encephalitis, ophthalmia and abortion (7–9).

Previous research has demonstrated that direct contact with cats is not considered a primary risk for human infection due to the short duration of oocyst shedding (10). However, cats could continue to shed millions of oocysts for about 1–2 weeks in their lifetime, which can survive for months in the environment and become a major contributor to human infection (11, 12). With the improvement of living standards, there is a raise in the number of urban cats (13). An increasing number of studies have reported the surveys of *Toxoplasma* seroprevalence and molecular detection in the world, including some areas of China (3, 11, 14). It is well known that seroepidemiological studies are useful for indicating infection in an area and a special population of cats, which can be used to assess the infection risk for the definitive and intermediate hosts (15). In recent years, seroprevalence of *Toxoplasma* in cats has been reported to distribute in 5 to 40% in mainland China, yet seroprevalence can be as high as 100% due to sample size and geography (16, 17). Molecular diagnostics of toxoplasmosis were generally based on detection of specific DNA sequences, and the detection of *T. gondii* DNA in cat feces was generally considered as the presence of oocysts. An earlier serological survey revealed that *T. gondii* IgG antibodies in pet dogs and pregnant women were 21.6 and 29.2%, respectively, which were speculated to be associated with the existence of a high density of urban cats, exposing people and animals to an elevated density of oocysts (18). However, there is still limited information on the prevalence of *T. gondii* in urban cats in Kunming, the capital of Yunan, Southwest China, and the risk factors of toxoplasmosis in cats in this region also remain unclear.

In the present study, the serological and molecular survey of *T. gondii* infection were determined in urban cats in Kunming, Southwest China. In addition, we analyzed the evolutionary relationship of the isolate of *T. gondii* in current study, and evaluated the associated risk factors exposure to the parasite, which could provide public health workers with useful information for the prevention and control of the zoonosis, and for the protection of human health.

## Materials and methods

### Sample collection

A total of 231 serum and fecal samples were collected from the leg veins of cats between October 2022 and September 2023 in Kunming, Southwest China, with the geographical coordinates of 25°02′47″N and 102°42′34″E, to detect the seroprevalence and fecal DNA of *T. gondii* in urban cats. The blood samples were left at room temperature for 1 h, followed by centrifugation at 3000 rpm for 10 min to separate the serum, which was stored at −20°C for subsequent ELISA analysis. All feces from cats were harvested from rectum, and the fresh feces were immediately sent to laboratory for DNA determination.

### Serological assay

The ELSIA assays were conducted to detect anti-*T. gondii* antibodies in the serum samples, and the methods were described previously (19, 20). Briefly, freshly egressed *T. gondii* ME49 tachyzoites were collected and lysed by sonication (21). Subsequently, the lysate was centrifuged at 12000 rpm for 10 min at 4°C, and the soluble *Toxoplasma* antigen (TSA) were determined by BCA Protein Assay Kit (Beyotime Biotechnology, Beijing, China). Next, the ELISA assays were conducted to investigate anti-*T. gondii* antibodies in urban cats (20). The 10 μg/mL TSA was diluted in coating buffer (0.05 M Carbonate–Bicarbonate, pH 9.6) at 4°C overnight to perform indirect ELISA analysis, and the serum samples diluted at 1:100 were added to each well. After washing, the HRP conjugated anti-cat IgG secondary antibodies (Solarbio, Rabbit-anti-Cat IgG: K0082R) were diluted 1:2000 and added to appropriate wells. The color was developed by the addition of substrate solution TMB, and the OD value was determined at 450 nm using a microplate reader. For the resulting judgment, the cut-off point of a positive sample was set to be at least two times higher than that of the negative sample.

### DNA isolation and PCR amplification

DNA was extracted from the feces of urban cats via Fecal DNA Isolation Kit (Tiangen, China) according to the manufacture’s protocols. Subsequently, the fecal DNA was amplified with the *Toxoplasma* RE 529-bp sequence specific primers (forward primer: 5’-TGACTCGGGCCCAGCTGCGT-3’ and reverse primer: 5’-CTCCTCCCTTCGTCCAAGCCTCC-3’) by PCR. The primer pairs and PCR temperature cycling conditions were in according to a previous study (22, 23). The PRC reactions were carried out with a total of 25 μL reaction volume that contained 15 ng of template DNA, 12.5 μL of 2× Taq PCR mix (Vazyme Biotechnology Co., Ltd., Nanjing, China), 1 μL of each primer, and 9.5 μL of ddH_2_O. PCR program was initiated at 95°C for 5 min, followed by 35 cycles at 95°C for 45 s, 60°C for 45 s, 72°C for 45 s and finished with 72°C for 5 min. Extraction blanks (containing no DNA) were included as negative controls to test for contamination, and DNA was extracted from *T. gondii* type II strain as the positive control. Finally, the amplified products were loaded on 1.5% agar, and then evaluated under UV [GeL Logic 510-Imaging System (SHST, China)]. The length of the DNA fragment expected to be amplified was approximately 529 bp.

### DNA sequencing and phylogenetic analysis

The PCR product was sequenced (Sangon Biotech, Shanghai, China), and sequence analysis of RE 529-bp gene was performed to determine the *T. gondii* strain. The sequences were analyzed by Basic Local Alignment Search Tool (BLAST), and compared with those of available sequences in the GenBank (24). Phylogenetic analysis was performed using MEGA software (Version: 11) through the neighbor-joining method with 1000 bootstraps to accomplish several sequence alignments (25).

### Statistical analysis

The prevalence and 95% confidence intervals per pathogen species were calculated using the Vassarstats program.^1^ Differences in *T. gondii* prevalence for different variables such as gender, breed, food, water, age and lifestyle were analyzed using a chi square test. The differences were considered statistically significant when the resulting *p* < 0.05.

## Results

**Figure 1:**
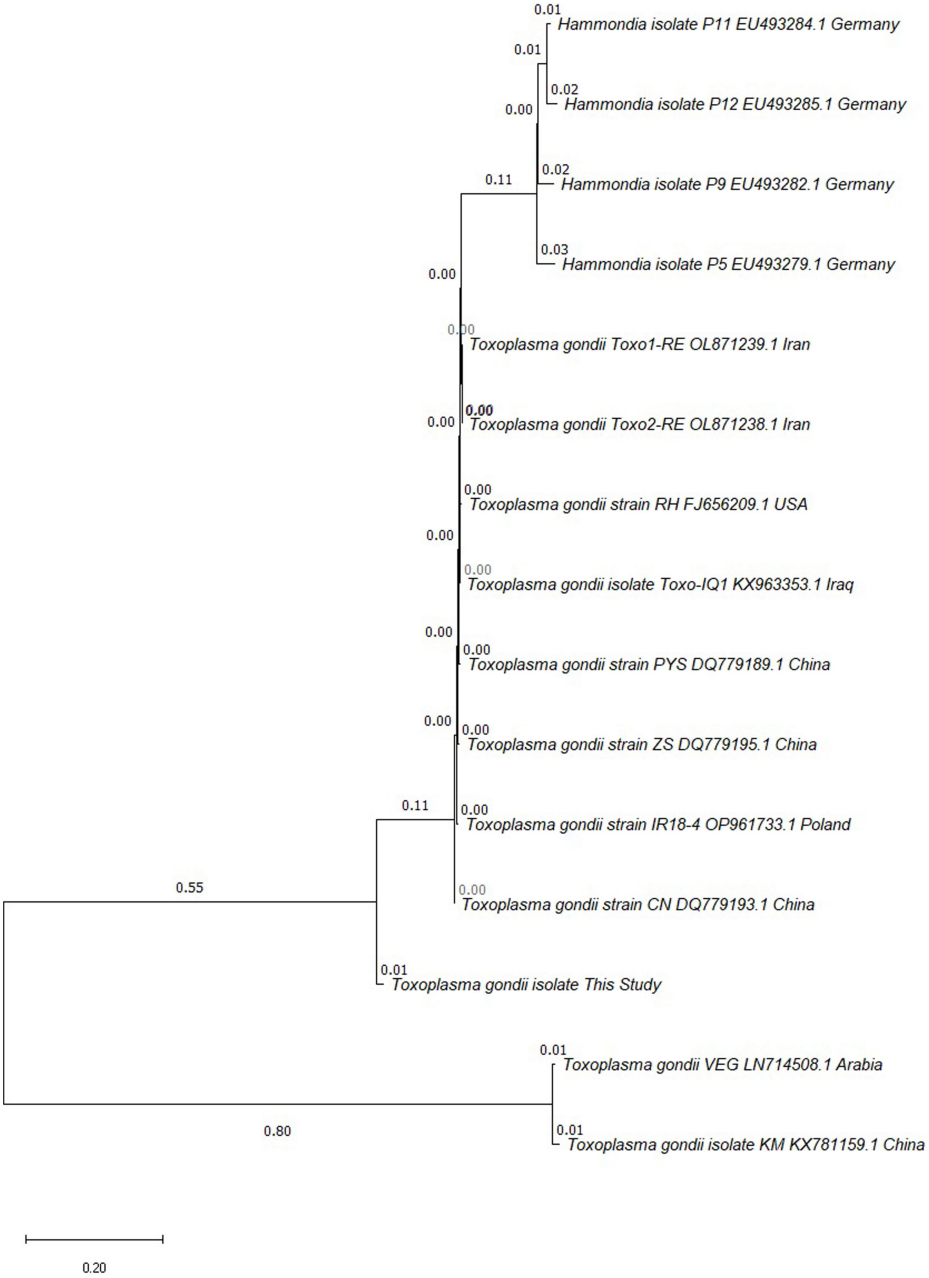
Phylogenetic tree analysis based on repetitive RE 529-bp gene partial sequence was used to confirm the identification of *T. gondii* isolates by neighbor-joining method.

Of the 231 examined samples, 168 (72.7%) were positive for *T. gondii* antibodies, and only one (1.4%) sample examined by RE 529-bp PCR showed a positive for *Toxoplasma* DNA (Table 1 and Supplementary Figure S1). The PCR product of RE 529-bp gene was sequenced and the result was analyzed by BLAST program. The obtained oligonucleotide sequence was submitted to Genbank database, and the evolutionary tree was constructed by MEGA software. The alignment results revealed that the homology level of *T. gondii* repetitive sequencing products compare to other strains is varies ranging from 79 to 91%. The phylogenetic analysis revealed that the isolate was more closely linked to *T. gondii* strain CN, an isolate from China (DQ779193.1) (Figure 1).

**Table 1:** Prevalence of *Toxoplasma gondii* antibodies and antigens in domestic cats.

IgG			PCR		
No. of tested	Positive	Prevalence% (95% CI)	No. of tested	Positive	Prevalence% (95% CI)
231	168	72.7 (66.7–78.1)	74	1	1.4 (0.2–7.3)

Next, we analyzed the common potential risk factors for the infection of cats with *T. gondii*, including gender, breed, food, water, age and lifestyle. The results suggested that there was no significant association between gender or breed and *T. gondii* positivity (Table 2). Nevertheless, the seropositivity was found to be related to the diet of cats, with a higher positive rate in cats fed with animal organs (61.8%) and tap water (88.0%) than in cats fed with homemade cooked food (8.7%) and boiled water (52.0%). Furthermore, the seroprevalence of *T. gondii* was significantly associated with age of cats and increased with the ages of cats (Table 2). Unlike other studies, the present study identified no significant relationship between lifestyle and *T. gondii* positivity, although stray cats were more likely to be exposed to *T. gondii* oocysts.

**Table 2:** Analysis of factors associated with *Toxoplasma* positive.

Variable	Diagnostic test	No. of cats (%)	No. of positive	Prevalence%	95% CI	*p* value
Gender						
Male	IgG	131	91	69.5	61.1–76.7	0.203
	PCR	52	1	1.9	0.3–10.1	
Female	IgG	100	77	77.0	67.9–84.2	
	PCR	22	0	0		
Breed						
Mixed breed	IgG	52	39	75.0	61.8–84.8	0.676
	PCR	17	0	0		
Thoroughbred	IgG	179	129	72.1	65.1–78.1	
	PCR	57	1	1.8	0.3–9.3	
Food						
Commercial cat food	IgG	174	145	83.3	77.1–88.1	<0.001
	PCR	66	1	1.5	0.3–8.1	
Homemade cooked food	IgG	23	2	8.7	2.4–26.8	
	PCR	6	0	0		
Animal organs	IgG	34	21	61.8	45.0–76.1	
	PCR	2	0	0		
Water						
Tap water	IgG	133	117	88.0	81.4–92.5	<0.001
	PCR	29	1	3.5	0.6–17.2	
Boiled water	IgG	98	51	52.0	42.3–61.7	
	PCR	45	0	0		
Age						
≤1	IgG	101	61	60.4	50.7–69.4	0.002
	PCR	23	1	4.4	0.8–21.0	
1 < Age < 2	IgG	39	35	89.7	76.4–95.9	
	PCR	31	0	0		
2 ≤ Age < 10	IgG	87	69	79.3	69.7–86.5	
	PCR	19	0	0		
≥10	IgG	4	3	75.0	30.1–95.4	
	PCR	1	0	0		
Lifestyle						
Domestic cats	IgG	211	154	73.0	65.1–77.2	0.774
	PCR	51	1	2.0	0.4–10.3	
Stray cats	IgG	20	14	70.0	48.1–85.5	
	PCR	23	0	0		

## Discussion

*T. gondii* is a widespread zoonotic pathogen, and cats are the ultimate host of *T. gondii* and can excrete numerous oocysts. Therefore, cats play a crucial role in the transmission of *T. gondii*, and the serological and molecular survey of *T. gondii* infection in cats are essential for the development of toxoplasmosis prevention and control. Previous studies have reported the seroprevalence of *T. gondii* in cats in Southwest China, however, there was lack of information about the prevalence of toxoplasmosis in urban cats in Kunming, Southwest China (3, 26). In the present study, a total of 231 serum and 74 fecal samples of cats were detected, with positivity of 72.7 and 1.4%, respectively, which were the highest in China report so far. In China, seroprevalence of *Toxoplasma* infection in cats has been reported in some provinces, with a distribution of infection rates ranging from 2.5 to 60.0%, for an overall infection rate of 20.2% (14). Previous surveys reported varying seroprevalence of *T. gondii* infection in pet cats in Southwest China: 63.16% in Guizhou (26), 13.0% in Sichuan (3), and 12.8% in Chongqing (3). In addition, an earlier survey mentioned that the seroprevalence of *T. gondii* infection in cats in Kunimg was as high as 50.3%, although this data has not yet been published (18). Compared with the previous sero-epidemiological studies form these reported provinces, the frequency of *Toxoplasma*-seropositive cats in our study was significantly higher. The difference in seroprevalence for *T. gondii* may be relate to ecological and geographic factors, as well as the welfare status of urban cats in the areas. Another possible reason for the high *T. gondii* seropositivity is the dietary habit of eating raw meat in Kunming region, and the urban cats in Kunming are likely to be fed with undercooked food contaminated with cysts. Taken together, above data indicated that toxoplasmosis was widely spread cats in the region.

As the only terminal host of *T. gondii*, the oocysts excreted in the feces of cats are a huge threat to the environment and other animals (27). The molecular investigation of *T. gondii* infection is still limited, and our study is the first to report the *Toxoplasma* infection in cat feces in Kunming. The molecular prevalence of *T. gondii* infection in Kunming is 1.4%, which was different from 0% in Qinghai (14) and 52.63% in Guizhou (26). The high seropositivity rate of *T. gondii* but low PCR positivity rate in urban cats in Kunming is consistent with the results of some previous studies. Prevalence of anti-*T. gondii* antibodies was 14.8%, and only 0.7% positive rate for PCR (28). The seroprevalence of *T. gondii* infection in cats was as high as 41.4%, whereas the DNA positivity rate was 0% (14). It is generally believed that the definitive host sheds oocysts for about 2 weeks at the time of initial infection and thereafter no longer sheds oocysts upon secondary infections, due to long-term immunization preventing oocyst shedding (29, 30). Therefore, seropositivity of *T. gondii* does not mean that the cat is excreting oocysts, which requires further measurement by PCR (30, 31). In addition, it has been suggested that the detection of *T. gondii* DNA in the feces of cats does not necessarily imply oocyst shedding, and there may exist bradyzoites from prey passing through gut, which are also infective (32, 33). The strain identified in current study was closely related to the isolate from Guangdong area, while more distantly related to typical strains (types I and III strains), and to the strain previously reported in cat feces in Kunming (34).

Common potential risk factors for *T. gondii* infection in cats include gender, breed, food, water, age and lifestyle (35, 36). Similar to other studies, the present study also confirmed that gender was not a key factor for *Toxoplasma* infection (3, 37). However, there was no significant difference in *T. gondii* seropositivity between mixed breed and thoroughbred cats, which may imply that there was no preference for different cat breeds in Kunming area. In terms of age, the seropositive rate of *T. gondii* was 60.4% in cats under 1 year of age, while the seropositive rate was much higher in cats older than 1 year of age, which may be related to the increased probability of exposure to *T. gondii* through food, water, and outdoor activities as cats grow older (38). Furthermore, the lower seropositivity of *T. gondii* in cats fed with homemade cooked food and boiled water suggested that cats were less susceptible to *T. gondii* in the feeding model. However, fecal *Toxoplasma* positive rate was higher in younger cats, suggesting that cats have previously been infected with *T. gondii*, and that they no longer secrete oocysts with age, which is consistent with the serologic results. In addition, this study also found that there was no significant correlation between the seropositive rate of *T. gondii* and lifestyle, which was different from some previous reports (3, 11), suggesting that urban cats in the Kunming area are raised in a special way, especially domesticated cats, whose seropositive rate of *T. gondii* was not significantly different from that of stray cats, suggesting that pet owners in this area may often feed their cats undercooked meat or contaminated water. It is worth drawing attention to the fact that the high seropositive rate of domesticated cats, indicating that a large number of *T. gondii* oocysts may be present in the environment of the region, which may have contaminated the water source in the region.

In conclusion, our study reveals that the infection rate of *T. gondii* in cats in Kunming area is high, which implies there is a hidden danger of public health safety. As a common pet in people’s life, cats carry pathogens that pose a threat to human health. Therefore, the investigation and analysis of *T. gondii* infection in cats is of great significance for avoiding human infections and formulating scientific and reasonable strategies to control toxoplasmosis. More measures need to be taken in order to protect the public health. The proper disposal of cat litter, and feeding cats with cooked food and boiled water as much as possible to reduce the oocyst burden are recommended. In addition, given the high seroprevalence of *T. gondii* IgG antibodies, it is necessary to perform a screening test and determine the IgG antibody titer in pregnant women and immunocompromised humans in Kunming, Southwest China.

## Data availability statement

The original contributions presented in the study are included in the article/Supplementary material, further inquiries can be directed to the corresponding authors.

## Ethics statement

The animal studies were approved by the Life Scientific Ethic Committee of Yunnan Agricultural University (Approval number: 202207006). The studies were conducted in accordance with the local legislation and institutional requirements. Written informed consent was obtained from the owners for the participation of their animals in this study.

## Author contributions

CY: Data curation, Formal analysis, Investigation, Methodology, Project administration, Writing – review & editing. SL: Methodology, Project administration, Writing – review & editing. CT: Data curation, Investigation, Methodology, Project administration, Writing – review & editing. JYu: Data curation, Writing - review & editing. MY: Data curation, Writing - review & editing. LG: Data curation, Writing - review & editing. LB: Data curation, Writing - review & editing. XL: Project administration, Writing - review & editing. JYa: Data curation, Funding acquisition, Methodology, Writing – original draft, Writing – review & editing. KJ: Validation, Writing – review & editing, Funding acquisition, Methodology, Writing – original draft.

## Footnotes

^1^ http://vassarstats.net/index.html

## Supplementary material

### supplementary FIGURE S1

PCR products of isolated *T. gondii* from cat feces based on RE 529-bp gene. M, 3000 bp molecular weight marker; P, positive control; N, negative control; lanes 1-22, PCR product of RE 529-bp gene.
