# Supplementary figures and images for: Serological and molecular survey of Toxoplasma gondii infection and associated risk factors in urban cats in Kunming, Southwest China
Source: Front Vet Sci. 2024 Jun 18;11:1393236. doi: 10.3389/fvets.2024.1393236 (PMC11218566; doi:10.3389/fvets.2024.1393236)

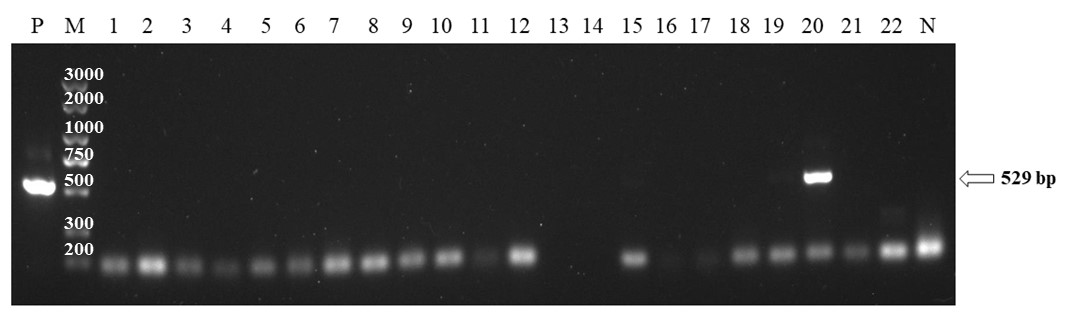

Supplement: Supplementary file 1 [file Image_1.JPEG]
